# Dr. Patterson’s Valedictory Address

**Published:** 1844-05

**Authors:** 


### DR. PATTERSON'S VALEDICTORY ADDRESS.

Professor Patterson, of the Pennsylvania Medical College, delivered a sensible and appropriate address to his graduating class at the close of the last session, of which we have had the pleasure to receive a copy. We will add that we experienced much pleasure in reading it. It is the production of a gifted and cultivated mind, characterised by modesty and a love of truth. The thoughts are occasionally excellent beyond the average of such discourses. Take the following as examples:

"No class of men appear more backward than the members of our profession to admit the correctness of the position so stoutly contended for by a late author, that he who prints, for any other reason than because he has something in him which deserves to be printed, sins against the peace and comfort of the community. In these latter days of book-making, too many, inspired by the mere lucre of gain and the poor ambition of seeing their names printed on a title-page, have poured out treatise upon treatise, compiled and most unskilfully collated from foreign authors of all grades of authority, and in which you will find an attempt to extract the original matter as hopeless and unprofitable as the process of Gulliver's philosopher for obtaining moon-shine from cucumbers. Under these circumstances you would of course, err in giving equal credence to all authors, without discrimination. This fact also renders inexcusable those who state no settled opinions of their own, but merely give the conflicting views of others; balancing one array of names against another, as though they were counting the black and white balls in a ballot-box, without giving the student or the man of small library a clue to the authority properly attaching to any one of them. But while endeavoring to distinguish authoritative writers from others, you must be equally guarded against falling into the contrary error, an implicit reliance upon the dictum of any one man. The rule here, as elsewhere, is that the truth makes the authority, and not the authority the truth. Let your reverence for the individual be ever so great, you must bear in mind that our science is still progressive, and that no man has yet reached its ultimate truths, or can say to its swelling tide, 'Thus far shalt thou come and no farther.'"

And again:

"Never forget that you are not the master of Nature, but her humble attendant and assistant; that you are not the source of law to the vital economy, but merely the servant to aid the operation of its laws. The forces of the living organism are in a great measure beyond your control. Be cautious how you disturb them. Never look on a disease as some corporate entity which you can demolish at a blow. I would inculcate upon you no indolent Expectancy, no sluggish "meditation upon death," but I would advise you to be careful lest you tamper too freely with this complicated mechanism and its occult forces. I fear that the appeal of the quaint emblemist, Quarles, is addressed properly to many even in our day:

"Hold thy hand, health's dear maintainer;
Life perchance may burn the stronger;
Having substance to maintain her,
She untouched may last the longer.
When the artist goes about
To redress her flame, I doubt
Oftentimes he snuffs it out.

"But to the man of correct physiological views, who is acquainted with the laws of the organization upon which his medicinal agents operate, no such appeal need be made. He knows his position and his duty, and as he sits by to replenish the oil and tend the flame of the lamp of life, he can never imagine himself the source and fountain of its elemental heat and light."

This farewell address seems to have been delivered by Professor Patterson to his first class. We shall expect to hear of him again, as one who has won a name among the many renowned medical teachers of Philadelphia. Y.

